# Editorial: Cancer in domestic, exotic and wild animals: new horizons in tumorigenesis, diagnosis, prognosis and therapeutics through comparative oncology

**DOI:** 10.3389/fvets.2025.1702872

**Published:** 2025-10-17

**Authors:** Yasunaga Yoshikawa, Isabel Pires, Leonardo Leonardi

**Affiliations:** ^1^Laboratory of Veterinary Biochemistry, School of Veterinary Medicine, Kitasato University, Aomori, Japan; ^2^Animal and Veterinary Research Center (CECAV) University of Trás-os-Montes and Alto Douro, Vila Real, Portugal; ^3^Department of Veterinary Medicine, University of Perugia, Perugia, Italy

**Keywords:** cancer, comparative oncology, dog, elephant (*Loxodonta africana*), giant pandas (*Ailuropoda melanoleuca*), roe deer (*Capreolus capreolus*), human, One Health

Significant advances in understanding cancer biology have been made in recent years, leading to increasingly accurate and early diagnosis and specific and effective therapeutic treatments. Advances in the identification of increasingly sophisticated prognostic markers have enabled veterinarians to predict the course of many neoplastic diseases, improving the quality of clinical and therapeutic approaches and the rate of recovery for many animals affected by cancer.

Supporting these scientific advances and the fundamental and exciting findings of recent years, biomolecular and comparative studies have also been conducted across multiple animal species, including humans. These studies have allowed for the biological and genetic characterization of numerous tumors, further strengthening the importance of scientific approaches aimed at a “One Health” assessment. The comparative study of cancer in different animal species, including domestic, exotic, and wild animals, can provide valuable information of various types and degrees, with considerable potential for improving diagnostic, prognostic, and therapeutic approaches. Recent studies have shown that even particularly tumor-resistant animals, such as naked mole rats, blind mole rats, elephants, and whales, have attracted increasing attention from researchers, with the aim of characterizing the most intimate mechanisms of disease resistance in these species and breeds (1). These cancer resistance mechanisms observed in these species could offer new strategic approaches for anticancer treatments in humans, as well as in domestic and exotic animals. Therefore, by examining both tumor-prone animals closest to humans, such as dogs and cats, and tumor-resistant species, comparative oncology explores the similarities and differences between human and animal cancers, contributing to the development of new diagnostic tools, therapies, and preventive strategies that advance both human and veterinary medicine.

These Research Topics can represent important comparative platforms in the field of oncology, representing a new intersection between medical sciences, veterinary sciences, comparative oncology, and wildlife and environmental conservation. Our goal is to create a dynamic channel for sharing cutting-edge scientific knowledge and identifying novel diagnostic, therapeutic, and preventive strategies in the largely unexplored realm of multispecies comparative oncology. This Research Topic presents a broad spectrum of contributions—three original research articles, two short reports, one clinical trial article, and six case reports—covering diverse species, including tumor-prone animals, tumor-resistant species, and wild animals. The following paragraphs summarize the key findings reported by several authors in their nine manuscripts that comprise this Research Topic.

Dogs are among the most commonly tumor-prone species in veterinary medicine. This Research Topic includes several studies focusing on canine tumors. Maniscalco et al. proposed a new scoring system that effectively distinguishes dogs with favorable-prognosis hepatoid gland tumors from those with worse prognoses, thus supporting histological diagnosis. Chu et al. evaluated treatment outcomes in dogs with transitional cell carcinoma (TCC) through a retrospective analysis and suggested that metronomic chemotherapy with chlorambucil was well-tolerated and can be considered as a single-modality treatment or as an adjunct to conventional chemotherapy. Power et al. demonstrated the potential of a novel iron-related metabolic target in canine osteoblastic osteosarcoma using pathological techniques. These results suggest that targeting iron metabolism may represent a novel therapeutic strategy. Tsumoto et al. described a recently introduced method using flow cytometry (FCM) to rapidly detect survivin expression and localization in needle biopsy specimens without anesthesia. The technology could support cancer vaccines and targeted therapies, helping to improve veterinary care through the “one-day first” program. Clinical data were also presented. Xia et al. described a clinical trial of immunotherapy with a vaccine based on dendritic cell/tumor cell fusion and demonstrated its safety. Furthermore, two case reports have indicated the utility of PET in canine cancer (Seok and Lee; Wang et al.). They would be valuable in highlighting the diagnostic and prognostic potential of PET imaging, particularly in detecting metastatic spread, guiding surgical planning, and monitoring treatment response. We also included the rare case of canine cancer. In addition, Pop et al. described a rare ossifying fibroma (OF) of the zygomatic bone in a 9-year-old Hungarian Vizsla.

This Research Topic also features studies on elephants, a non-model but cancer-resistant species that provides valuable insights into anticancer mechanisms. Kitano et al. reported on the responses of elephant cells to interstrand crosslinks in comparison with human cells.

Furthermore, we also included cancer studies focused on wild animals. Xiong et al. (a) and Xiong et al. (b) reported two clinical cases in giant pandas: one described oral fibrosarcoma and the other mandibular osteosarcoma. Vengušt et al. described a rare case of neuroendocrine carcinoma in the nasal cavity of a roe deer, highlighting its histopathological and immunohistochemical characteristics.

Overall, this Research Topic highlights current cancer research in a wide range of animal species, providing fundamental studies and diagnostic and therapeutic tools. It also focuses on comparisons between animals and humans, offering insights into recent advances in comparative oncology.

## References

[1] Seluanov A Gladyshev VN Vijg J Gorbunova V. Mechanisms of cancer resistance in long-lived mammals. Nat Rev Cancer. (2018) 18:433–41. 10.1038/s41568-018-0004-9 29622806 PMC6015544

